# The Biology and Function of Tissue Inhibitor of Metalloproteinase 2 in the Lungs

**DOI:** 10.1155/2022/3632764

**Published:** 2022-12-31

**Authors:** Louis Costanzo, Brian Soto, Richard Meier, Patrick Geraghty

**Affiliations:** Department of Medicine, State University of New York Downstate Health Sciences University, 450 Clarkson Avenue, Brooklyn, NY 11203, USA

## Abstract

Tissue inhibitors of matrix metalloproteinases (TIMP) are a family of four endogenous proteins that primarily function to inhibit the activities of proteases such as the matrix metalloproteinases (MMP). Altered MMP/TIMP ratios are frequently observed in several human diseases. During aging and disease progression, the extracellular matrix (ECM) undergoes structural changes in which elastin and collagens serve an essential role. MMPs and TIMPs significantly influence the ECM. Classically, elevated levels of TIMPs are suggested to result in ECM accumulation leading to fibrosis, whereas loss of TIMP responses leads to enhanced matrix proteolysis. Here, we outline the known roles of the most abundant TIMP, TIMP2, in pulmonary diseases but also discuss future perspectives in TIMP2 research that could impact the lungs. TIMP2 directly inhibits MMPs, in particular MMP2, but TIMP2 is also required for the activation of MMP2 through its interaction with MMP14. The protease and antiprotease imbalance of MMPs and TIMPs are extensively studied in diseases but recent discoveries suggest that TIMPs, specifically, TIMP2 could play other roles in aging and inflammation processes.

## 1. Introduction

Tissue inhibitors of metalloproteinases (TIMPs) 2, one of four members of the TIMP family, govern pericellular proteolysis of the extracellular matrix (ECM) and cell surface proteins through inhibition of matrix metalloproteinases (MMP) activity and are an important component in the regulation of ECM turnover [1]. MMPs consist of a family of enzymes that are directly responsible for the degradation of the ECM but also play a part in the pathogenesis of multiple diseases, including lung diseases [2]. Importantly, MMPs do play a major role in other physiologic processes, such as cell migration and angiogenesis [3]. Thereby, MMPs facilitate the maintenance of the cellular environment during embryogenesis, morphogenesis, and continuous tissue remodeling [4]. They function as endopeptidases, which break apart amino acids within a molecule or exopeptidases, which cleave the penultimate peptide bond. Their activity is regulated by the expression of genes, zymogenic conversion, and the presence of proteolytic inhibitors. Despite differences in affinity, all TIMPs are generally viewed as broad-spectrum MMP inhibitors. TIMP1 has the highest affinity for MMP9, which is implicated in immune cell function and fibrosis in cardiovascular disease [5]. TIMP3 exhibits the strongest interaction with low-density lipoprotein receptor-related protein 1 (LRP-1), a disintegrin and metalloproteases (ADAM)10, angiotensin II type 2 receptor (AT2R), MMP9, and MMP13 [6]. It also forms a complex with MMP2, though TIMP2 is considered a more potent inhibitor of MMP2 [7]. Finally, TIMP4, a key modulator of MMP9 and PAR-1 activity [8], may decrease pro-MMP2 activation when coexpressed with TIMP2 [9].

TIMPs inhibit the activities of these MMPs, as well as several ADAMs and ADAMs with thrombospondin type I motifs (ADAMTSs) [10]. The extracellular localization of TIMPs is controlled by LRP1-mediated endocytosis, which dynamically regulates pericellular TIMPs, MMPs, and ADAMs [11]. TIMPs also are known to exert diverse biological functions independent of their ability to inhibit metalloproteinases. However, the majority of TIMP literature primarily focuses on their antiprotease functions, which we will outline before evaluating other possible functions of TIMP2, such as modulation of cell growth, proliferation, inflammation, migration and inhibition of cellular invasion, tumorigenesis, metastasis, angiogenesis, and cellular aging.

## 2. TIMP2: Function, Regulation, and Structure

TIMP2 is known to form noncovalent high-affinity complexes with the following MMPs: MMP1, MMP2, MMP3, MMP7, MMP8, MMP9, MMP10, MMP13, MMP14, MMP15, MMP16, and MMP19 [12–14]. More specifically, an MMP14 molecule binds to TIMP2 to form a complex which acts as an adaptor for pro-MMP2. The binding of this complex to pro-MMP2 occurs via the interaction of the TIMP2 C-domain and the MMP2 hemopexin domain. Once the tertiary complex is formed, a second MMP14 molecule can act as an activator to cleave pro-MMP2 and release the active MMP2 [15–17]. MMP2 is highly expressed in numerous cancers including breast cancer [18], cervical cancer [19], bladder cancer [20], gastric cancer [21], and lung cancer [22], which makes MMP2 an important anticancer therapy target. Not only does MMP2 play a role in excessive ECM degradation allowing for tumor cell metastasis and invasion [23] but MMP2 is also implicated in cancer advancement through cellular apoptosis, proliferation, and angiogenesis [2, 24, 25]. MMP2 also plays a role in skin cancer, as B16 melanoma cells that express MMP2 have slower kinetics in *Tlr*2^−/−^*Tlr*4^−/−^ mice, which suggests that MMP2 overexpression could contribute to tumor growth [26].

Depending on the cell line and specific inhibitor, TIMPs may have either inhibitory or stimulatory effects on cell cycle. Both TIMP1 and TIMP2 can activate MMP2 and MMP9 by binding MMP14 and ADAM 10 receptors on the cell surface [17]. More specifically, the binding of TIMP2 to MMP14 allows cleavage of pro-MMP2 by a free MMP14 molecule, as active MMP2 can cleave pro-MMP9 leading to dual gelatinase activity. TIMP2 can also bind *α*3*β*1 integrin at the cell surface leading to SHP-1 activation which promotes cell cycle arrest through nuclear localization of p27 [17]. Therapeutic agents may target pro-MMP2 activation which occurs in the presence of MT1-MMP through extracellular c-Src tyrosine kinase phosphorylation of TIMP2 [27]. Additionally, HT-1080 fibrosarcoma cells have increased cyclic adenosine 3′,5′-monophosphate (cAMP) levels when treated with purified recombinant TIMP2, suggesting cell-surface binding of the TIMP2-pro-MMP2 complex and activation of a second messenger system [28]. *In vitro* studies suggest that the activation of proMMP-2 occurs through formation of a trimolecular complex involving MMP14, TIMP2, and proMMP2 at the cell surface [29]. Pro-MMP2 also forms a tight complex with TIMP2 in the fibroblasts of mice, with *Timp*2-deficient mice incapable of activating pro-MMP2 [30]. The loss of TIMP2 did not adversely affect normal development, viability, or fertility on the C57BL/6 background but led to a significant decrease in activation of proMMP-2 [30]. Hence, TIMP2 does play a significant role in MMP2-dependent activation and expression. It should be noted that there are also MMP14-independent means of activating MMP2 [31].

The *TIMP2* gene, located on chromosome 17q25.3, contains 5 exons; all of which are separated by 4 introns of 54.8, 2.7, 9.1, and 1.7 kb [32]. However, only 2 transcripts of 1.2 and 3.8 kb are reported [33]. Ultimately, this gene codes for a proprotein initially produced in the endoplasmic reticulum with a size of 220 amino acids and a molecular mass of 24.4 kDa; though, when activated, the proprotein is cleaved to generate the mature TIMP2, which is 194 amino acids long with a mass of approximately 21 kDa [19]. TIMP2 is expressed in the majority of cell types throughout the body [7] and is the most widely expressed and observed TIMP in all normal tissues [34]. In the developing human fetus, TIMPs 1-3 are all detected in the fetal epithelium, while TIMP2 and TIMP3 are the only TIMPs detected in the pulmonary vascular endothelium [35]. In the fetal mouse, TIMP2 is expressed on days 11.5 and 13.5 [36]. Cloning of a 2.5 kb genomic portion of the human *TIMP*2 gene promoter sequence identified 519 base pairs of the 5′ flanking region; the initial codon is located at 432 base pairs [37]. The 5′ region has an increased G-C content and a noncoding TATA DNA sequence (AATAAAA) located at 23-37 base pairs upstream from a collection of transcription start points, several Sp1 motifs and one AP-2 motif, and an AP-1 sequence at position 590 to 583 from the start codon [37]. The position of the AP-1 sequence of TIMP2 is important, as treatment with 12-O-tetradecanoylphorbol-13-acetate only provokes a response within specific AP-1 consensus regions [38]. Methylation influences TIMP2 gene expression [39, 40], as well as TIMP1 [41] and TIMP3 [42]. Further, hypermethylation of the TIMP2 promoter causes transcriptional repression of TIMP2 levels in many types of tumors, including lymphoma [43] and prostate cancer [44], resulting in tumor metastasis through MMP activation.

TIMP2 mRNA differs from TIMP1 and TIMP3 by the selection of polyadenylation signal sites [38]. The mRNA stability of TIMP2 is reasonably long with its mRNA's half-life (approximately 48 hours) being longer than the human *β*-actin mRNA (20 hours) [38]. Therefore, changes in TIMP2 expression may not be dependent on mRNA stability. TIMP2 expression is negatively regulated by the microRNA, miR-410, and is associated with non-small-cell lung cancer progression [45]. TIMP2 is further downregulated by a feedback circuit consisting of HIF-1*α*/miR-210/HIF-3*α* [46]. Equally, miR106a suppresses TIMP2 expression resulting in enhanced cell proliferation, migration, and invasion in human gastric cancer cells *in vitro* [47]. Transcriptional suppression of TIMP2 in C57BL/6 J mice is mediated by CCAAT enhancer-binding protein alpha (CEBPA) and MYC following mono-2-ethylhexyl phthalate exposure [48].

**Figure 1:**
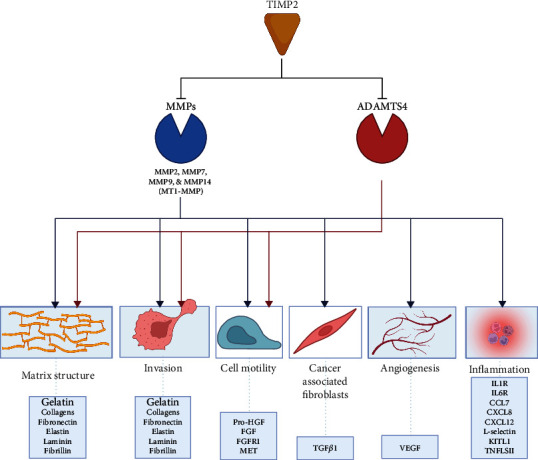
The primary established functions of TIMP2. TIMP2 inhibits several MMPs that cleave a range of substrates that impact structural and cellular aspects of tissue architecture. MMPs, membrane type 1 matrix metalloproteinase (MT1-MMP, also known as MMP-14), and ADAMTS break down ECM and structural proteins during cell invasion, motility, and tumor progression. Through proteolytic functions, TIMP2 affects a vast range of cellular processes in cells and the lung microenvironment. ADAMTS: a disintegrin and metalloproteinase with thrombospondin domains; CCL: CC-chemokine ligand; CXCL: CXC-chemokine ligand; FGF: fibroblast growth factor; FGFR1: FGF receptor 1; IL-1R: interleukin-1 receptor; KITL: KIT ligand; pro-HGF: prohepatocyte growth factor; TGF: transforming growth factor; TNF: tumor necrosis factor; TNFR: TNF receptor; TNFLS11: tumor necrosis factor ligand superfamily member 11; VEGF: vascular endothelial growth factor—created with http://BioRender.com.

The TIMP2 protein is nonglycosylated and has 2 distinct domains, an N-terminal domain containing 127 amino acids and a C-terminal domain comprised of 67 amino acids; each domain is stabilized by 3 disulfide bonds [49]. These disulfide loops located at the 1 netrin domain within the C-terminal facilitate binding to hemopexin-like domains of numerous members of the MMP-family, such as MMP2 [50]. Most of the interactions between TIMPs and their counterparts are made by the continuous ridge formed by the N-terminal five residues, C-S-C-S-P [7]. Single-site mutations in either the TIMP side chain (Cys1-Cys3 and Ser68-Cys72) or the AB loop (Ser31-Ile41) significantly change its affinity for MMPs, including MMP2 [51]. TIMP2 has an extended AB loop when compared to other TIMPs, which allows it to form a complex with MMP2 [52]. Carbamylation of the alpha-amino group of the N-terminal Cys1 may eventually inactivate TIMP2 [53]. TIMP2 activation can also be regulated by DNA methylation in cotyledon villous tissue, whereas investigators show that TIMP2 concentration increases when treated with lipopolysaccharide (LPS) but decreases when treated with methylation inhibitor 5-aza-2′-deoxycytidine (AZA) [54]. Therefore, modulation of TIMP2 should directly influence several MMPs, primarily MMP2 and MMP14, and impact several biological functions (See Figure 1).

There is also currently some controversy regarding the role that TIMP2 plays in cancers. Some data suggests that TIMP2 biological activity acts as a tumor suppressor. While there are various *in vitro* studies and clinical prognosis reports linking TIMP2 with tumor cell survival and proliferation. These oncogenic effects are contributed to TIMP2's interaction with MMP14 and subsequent downstream signaling [55]. This TIMP2 and MMP14 interactions are linked to PI3K/Akt and MAP kinase signaling activity [55, 56], but TIMP2 can also inhibit receptor tyrosine kinase signaling resulting in reduced proliferation and/or angiogenesis [57, 58].

## 3. TIMP2 Signaling beyond MMPs

TIMP2 expression also influences several responses, including those of mitogen-activated protein kinase (MAPK) [59] and *β*-catenin [60]. TIMP2 also plays several roles in various organs, such as growth-stimulatory activity [59], hippocampal function in aged mice [61], and the promotion of leukemia cell invasion [62]. Alternatively, *Timp*2 deficiency is associated with abnormal motor function [63] and cognitive dysfunction [64], as well as unfavorable outcomes in cancer development [65]. TIMP2 expression is induced by cytokines and chemokines, ROS, proliferation stimuli (such as *β*FGF and EGF), and differentiation factors (such as retinoic acid and NGF). Overexpression of TIMP2 significantly inhibited the production of nitric oxide (NO), tumor necrosis factor-alpha (TNF*α*), interleukin (IL) 1*β*, and ROS while increasing anti-inflammatory IL-10 production in mouse and rat microglia [66]. Alternatively, inflammatory responses can trigger TIMP2 expression or activity. Li et al. showed that TNF*α* and IL1*β* can regulate TIMP2 expression in cardiac cells [67]. TIMP2 levels are suppressed in cardiac fibroblasts by both cigarette smoke and aging [68]. IL-4 and IL-13 signaling are also linked to the expression of TIMP2 [69]. Lee and Kim showed that inhibition of TIMP2 in LPS-stimulated BV2 mouse microglial cells amplified the production of proinflammatory cytokines [66]. Further, overexpression of TIMP2 produces a neuroprotective effect via the suppression of microglial activation through anti-inflammatory Nrf2 and cAMP-response element-binding protein transcription factors [22], suggesting that TIMP2 may play a significant anti-inflammatory role, see Table 1 for a summary of TIMP2 functions in diseases.

**Figure 2:**
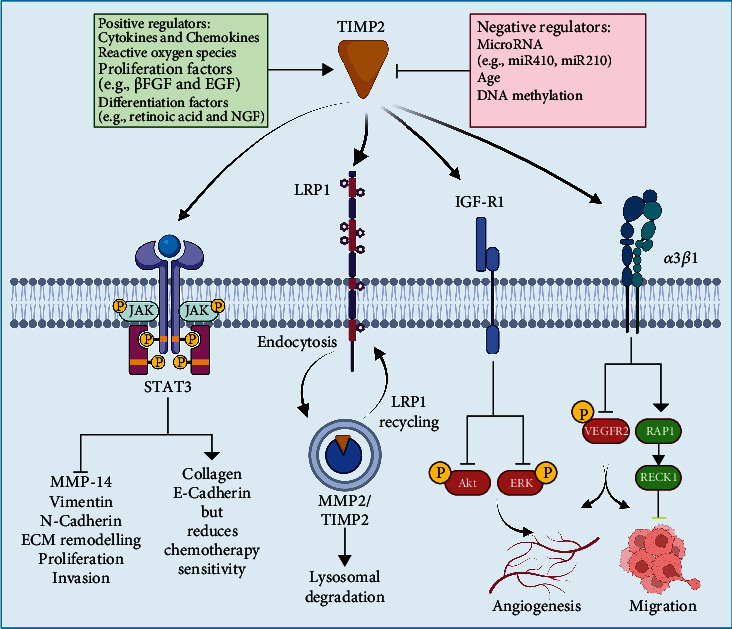
TIMP2 interacts with transcription factors and cell surface receptors. TIMP2 directly interacts with signaling molecules and transcription factors such as STAT-3, as well as cell surface receptors including *α*3*β*1 integrin, IGF1R, and LRP1 to alter signaling transduction—created with http://BioRender.com.

In addition to TIMP2's function on MMP2, TIMP2 directly interacts with cell surface receptors [70], such as the Janus kinase- (JAK-) signal transducer and activator of transcription (STAT) 3 [71]. TIMP2 also negatively regulates endothelial cell migration and invasion through *α*3*β*1 integrin [57]. TIMP2-deficient lung cancer cells grown in spheroids exhibit enhanced epidermal growth factor receptor (EGFR) signaling [72]. Putative targets for TIMP2 also include insulin-like growth factor 1 receptor (IGF1R) and LRP1/2 [73–75], see Figure 2 for a summary of TIMP2 interactions.

TIMP2 induces G1 cell cycle arrest by binding to human endothelial cells through integrin *α*3/*β*1. This TIMP2 association with G1 cell cycle arrest during the very early phase of cellular damage can serve as a biomarker to predict acute kidney injury *in vivo* [76]. In combination with insulin-like growth factor binding protein (IGFBP) 7, TIMP2 can block cyclin-dependent protein-kinase complex-mediated cell cycle promotion, thereby resulting in G1 cell cycle arrest to prevent the dividing of damaged cells [77]. TIMP2 also inhibits the mitogenic response of endothelial cells to growth factors, like vascular endothelial growth factor- (VEGF-) A and fibroblast growth factor-2 *in vitro* and *in vivo* [78]. TIMP2 could also bind to the *α*3*β*1 integrin heterodimer and thereby stimulate SHP1-mediated dephosphorylation of fibroblast growth factor receptor 1 (FGFR1) or EGFR. This would influence angiogenesis responses [57, 79]. Interestingly, TIMP2 is reported to inhibit tubulogenesis in aged human microvascular endothelial cell lines [80]. TIMP2 mediates growth arrest by inducing *de novo* synthesis of kinesin-related motor protein 1 (KIP1) and possibly leading to inhibition of cyclin-dependent kinase (CDK) 2 and CDK4 [81].

## 4. The Influence of TIMP2 Expression in Pulmonary Diseases and Associated Comorbidities

*Timp2-*deficient mice have no active lung MMP2 and do not exhibit any gross morphological or phenotypic effects under nonstressed conditions [30]. Within humans, increased levels of MMP2 and TIMP2 were detected in the bronchial alveolar lavage fluid of 48 patients with various types of lung cancers [82]. Moreover, MMP2 expression was elevated in the cytoplasm and membranes of those with poorly differentiated lung squamous cell carcinoma [83]. However, dysregulation between TIMP2 and MMP2 may lead to cellular dysfunction. Yao et al. showed that treatment with the toxic metabolite mono-(2-ethylhexyl) phthalate in rodent Sertoli cells causes an increase in MMP2 but a decrease in TIMP2 over time resulting in the interference of cellular processes like gametocytogenesis [84]. Additionally, TIMP2 influences MMP14 activity, and overexpression of MMP14 is observed in epithelial and stromal cells in non-small-cell lung cancer patients [85] and neurodegeneration and age-related changes [86]. MMP14 plays a crucial role in cancer migration and metastasis by ECM remodeling and cell motility, and MMP14 responses can be regulated by the scaffolding protein NEDD9 (neural precursor cell expressed developmentally downregulated 9) and TIMP2 levels [87]. The activity of MMP2 is regulated by MMP14 in combination with TIMP2 [15]. In a mouse myocardial infarction model, the heart tissue in *Timp2*^−/−^ mice is less dense with disorganized fibrillar collagen due to greater MMP14 activity [88].

Reduced expression of TIMPs is observed in senescent human fibroblasts [89], with senescence linked to several lung pathologies [90]. H_2_O_2_-induced premature senescent intervertebral disc cells have reduced expression of *TIMP1*, *TIMP2*, and *TIMP3* [91]. Within aortic sections of aged rats, the medial content of TIMP2 is significantly reduced in older animals [92]. TIMP2 inhibits the migration and apoptosis of macrophages and foam cells and inhibits atherosclerotic plaque development and destabilization [93]. TIMP2 deficiency accelerates adverse postmyocardial infarction remodeling due to enhanced MT1-MMP activity, despite a lack of MMP2 activation [88]. Overexpression of TIMP2 reduces brachiocephalic lesion area and stabilized plaques in an atherosclerosis model of ApoE^−/−^ mice on a high-fat diet [94]. These effects were linked to the inhibition of MMP14-dependent monocyte/macrophage infiltration and apoptosis.

Unlike TIMP1, loss of TIMP2 does not impact bleomycin-induced neutrophil recruitment [95]. Bleomycin-induced idiopathic pulmonary fibrosis (IPF), a disease characterized by chronic, progressive scarring of the lungs associated with a decline in respiratory function [96], induces the expression of both TIMP1 and TIMP2 in the alveolar and interstitial compartments [97]. Inhibition of GSK3*β* decreased the expression of MMP9, MMP2, TIMP1, and TIMP2 in inflammatory cells from bleomycin-treated mice [98]. Therefore, GSK3*β* could play a significant role in TIMP2 levels. SOCS1 can suppress the expression of TIMP2 as A549 cells and human embryonic lung fibroblasts [99]. In IPF patient samples, TIMP2 localizes to fibroblast foci, and TIMP2 colocalized with Ki67 in fibroblasts [100]. One IPF study suggested that TIMP2 in myofibroblasts contributed to the stable ECM deposition and the irreversible pulmonary structural remodeling, as TIMP2 was observed with MMP1, MMP2, and MMP9 in the regenerated epithelial cells covering intra-alveolar fibrosis [101].

Plasma levels of serpina3g, MMP9, TIMP1, and TIMP2 concentrations are reported higher in COPD patients compared to the controls, and higher levels are observed in COPD groups III and IV than in groups I and II [102]. This is surprising as others report an age-dependent reduction of plasma levels of TIMP2 [61]. The TIMP2 rs2277698 SNP is associated with overall and paraseptal emphysema and with FEV_1_/FVC ratio and MEF50 in a cohort of 951 construction workers [103]. COPD patients with elevated expression of both TGF*β*1 and TIMP2 have better pulmonary function test indices and reduced exacerbation frequency [104]. An immunohistochemical study of human lungs found an age-dependent increase of TIMP2^+^ cells in the lung, mostly determined in alveolar macrophages, bronchial epithelial cells, and mucosal fibroblasts [105]. There tends to be no gender-specific difference in plasma TIMP2 levels [106].

TIMP2 is expressed in postmitotic neurons and promotes neurite outgrowth and the differentiation of cells [107] because of cell cycle arrest through increased production of the cyclin-dependent kinase inhibitor p21Cip and decreased expression of cyclins B and D. In *in vitro* models, TIMP2 is expressed in *α*3 integrin-positive cells, suggesting that TIMP2-*α*3*β*1 integrin interactions participate in neurogenesis [63]. Interestingly, epithelial cell-specific deletion of *α*3 integrin prevents epithelial-mesenchymal transition in mice and protects against bleomycin-induced fibrosis [108]. Since TIMP2 enhances the expression of c-fos activation, this suggests a possible link to asthma as c-fos protein, and neuropeptide content in the lungs of asthmatic rats is related to asthma attacks [109]. However, little else is known about the role of c-fos activation in other pulmonary diseases. TIMP2 secreted by monocyte-like cells was identified as a potent suppressor of invadopodia formation in pancreatic cancer cells. TIMP2 may play a similar role within the lungs [110].

The link between TIMP2 and LRP1 could be relevant to pulmonary diseases. LRP1, a receptor involved in many cellular processes including cellular signaling, lipid homeostasis, and apoptotic cell clearance, is expressed in various tissues including the lungs [111]. This multitasking macroglobulin receptor mediates thrombospondin-dependent endocytosis of the pro-MMP-2-TIMP2 complex, as evidenced by the addition of receptor-associated protein to human fibrosarcoma HT1080 cells [112]. A genome-wide association study (GWAS) meta-analysis study of European subjects identified a significant association between lung function, specifically a reduction in the forced expiratory volume in 1 second (FEV1)/forced vital capacity (FVC) ratio to a single nucleotide polymorphism (SNP) that is mapped to an LRP1 intron [113]. Equally, smooth muscle cell-specific knockout of *Lrp1* alters the pulmonary function and airway responsiveness in mice [114]. Airway epithelial club cell knockout of *Lrp1* in mice influences lung inflammation and tissue damage and exacerbates smoke-induced lung disease due to a dysregulation of ROS and antioxidants [115].

A recent paper suggests that overexpression of TIMP2 or the stress-inducing gene, activating transcription factor 3 (ATF3), enhances autophagy activity with elevated p62 levels and the LC3BII/LC3BI ratio observed and decreased IL-6 and TNF-*α* levels in *Mycobacterium tuberculosis*-infected A549 cells [116]. This inflammation suppression was NF*κ*B-mediated. Therefore, TIMP2 may be playing a role in the lungs' anti-inflammatory responses.

## 5. Potential Antiaging Role of TIMP2

Recently, TIMP2 was linked to the overall survival in mice [117] and reduced aging within the brain [61]. At the time of writing this review, little is known about the potential of TIMP2 to prevent aging within the lungs. This is of importance as the initiation and progression of several pulmonary diseases are associated with aging. Time-dependent DNA damage, mutations, epigenetic alterations, accumulation of damaged and dysfunctional protein, altered energy metabolism oxidative stress, mitochondrial dysfunction, and senescence are frequently observed in aging [118]. Lung pathologies, such as IPF, COPD, and acute lung injury, increase considerably with age [119, 120]. Some of these changes occur at the molecular level and involve the accumulation of damaged and dysfunctional proteins, an increase in reactive oxidative species (ROS), expression of endoplasmic reticulum stress markers [121], epigenetic regulation [122], oncogene stimulation [123], mitochondrial dysregulation [124], and radiation-induced DNA destruction [125].

TIMP2 expression is sensitive to senescent responses, with lower expression levels of TIMPs observed in replicative senescent human fibroblasts and Werner syndrome fibroblasts [89, 126]. The disruption of the balance between the production of TIMPs and MMPs may contribute to aging and pulmonary disease [127]. Interestingly, age-related decline in TIMP2 protein is observed in hippocampal lysates, neurons of the subgranular zone, and hilar areas of the dentate gyrus of mice [61]. Systemic pools of TIMP2 are necessary for spatial memory in young mice, and treatment of brain slices with a TIMP2 antibody prevented long-term potentiation [61]. These investigators also observed that four 50 *μ*g/kg TIMP2 injections every second day elicit significant c-fos activation [61]. This suggests that c-fos could be critical in neuronal excitability and survival [128]. Equally, a recent article demonstrated that TIMP2 plays a role in fibroblast repair to prevent blood-brain barrier damage and hemorrhagic brain injury [129].

Wen et al. genetically engineered an attenuated strain of Salmonella as an anti-invasive vector for targeted delivery of TIMP2 into the striatum of U-87-malignant-glioma-bearing-BALB/cAnN nude mice and enhanced the survival rate by approximately 60% [117]. The expression of TIMP2 is also decreased in the patellar tendons of 3-year-old rabbits compared to 1-year-old rabbits [130]. However, TIMP2 expression and function must be further validated in pulmonary diseases as plasma levels of TIMP2 concentrations are reported higher in COPD patients compared to controls [102], and TIMP2 colocalizes with Ki67-positive fibroblasts in IPF patient lungs [100] with similar levels observed in the plasma of IPF and control patients [131]. Equally, we must be mindful that systemic levels of TIMP2 may not reflect the local lung levels and lung-specific signaling.

## 6. Concluding Remarks

The current literature suggests that we should consider TIMP2 not merely as an antiprotease but as a protein that could influence many signaling processes, including aging and inflammation processes. Systemic changes in blood levels of TIMP2 could have pulmonary implications. However, additional pulmonary-specific studies are required to explore the potential of TIMP2 signaling in the aging lung and its role in inflammation.

## Figures and Tables

**Table 1:** Known functions of TIMP2 and corresponding-associated diseases.

Functions	Diseases	Mechanism	Reference
Protooncogene induction	Asthma exacerbations Lung cancer	TIMP2 enhances c-fos expression; c-fos increased in lungs of asthmatic rats.	[14, 101]
Pro-MMP-2 activation	Squamous cell lung carcinoma	c-SRC tyrosine kinase induced pro-MMP-2 activation in the presence of MMP-14; MMP-2 found in high concentrations in SCLC.	[28]
Cell proliferation and inhibition of angiogenesis	Various types of lung cancers	Inhibition of VEGF-A and FGF-2 *in vitro* and *in vivo*. TIMP2 can bind *α*3*β*1 integrin heterodimer and stimulate SHP1-mediated dephosphorylation of FGFR1 or EGFR.	[70, 73, 82, 83]
Matrix stability and lung remodeling	IPF	TIMP2 colocalizes with Ki67+fibroblasts in IPF lungs; TIMP2 observed along with MMP1, 2, and 9 in regenerated epithelial cells covering intra-alveolar fibrosis.	[93, 94]
Inhibition of apoptosis	COPD	TIMP2 may inhibit apoptosis of macrophages and foam cells in airway epithelial cells, leading to decreased inflammation.	[88]
Extracellular matrix deposition	Acute lung injury	Bleomycin induces expression of TIMP2 in interstitial compartments.	[90, 92]
Antiaging	Dementia	Age-related decline in TIMP2 protein is observed in hippocampal lysates, neurons of the subgranular zone, and hilar areas of dentate gyrus of mice.	[14, 15]
	Tendinopathy	Age-dependent reduction of TIMP2 expression in rabbit patellar tendons	[130]
Anti-inflammatory	Systemic inflammatory response syndrome Neuroinflammatory disorders	TNF*α*/IL1*β* regulate TIMP2 expression in cardiac cells; inhibition of TIMP2 in microglial cells increase cytokine production. TIMP2 suppresses microglial activation through regulation of Nrf2 and cAMP-response element binding protein transcription factors.	[22, 69, 70]

## Abbreviations

IPF: Idiopathic pulmonary fibrosis, (IPF)
COPD: Chronic obstructive pulmonary disease
ECM: Extracellular matrix (ECM)
MMP: Matrix metalloproteinases
TIMP: Tissue inhibitors of metalloproteinases
SASP: Senescence-associated secretory phenotype
LRP1: Low-density lipoprotein receptor related protein 1
MT1-MMP: Membrane type 1 matrix metalloproteinase
ADAM: A disintegrin and metalloproteases
AT2R: Angiotensin II type 2 receptor
ADAMTS: ADAMs with thrombospondin type I motifs
cAMP: Cyclic adenosine 3′,5′-monophosphate
CEBPA: CCAAT enhancer-binding protein alpha
LPS: Lipopolysaccharide
AZA: 5-aza-2′-deoxycytidine
MAPK: Mitogen-activated protein kinase
ROS: Reactive oxygen species
NO: Nitric oxide (NO)
TNF: Tumor necrosis factor
IL: Interleukin
STAT: Signal transducer and activator of transcription
EGFR: Epidermal growth factor receptor
IGF1R: Insulin-like growth factor 1 receptor
GWAS: Genome-wide association study
FEV1: Forced expiratory volume in 1 second
FVC: Forced vital capacity
SNP: Single nucleotide polymorphism
IGFBP: Insulin-like growth factor binding protein
VEGF: Vascular endothelial growth factor
FGFR: Fibroblast growth factor receptor
KIP: Kinesin-related motor protein
CDK: Cyclin-dependent kinase
PPAR: Peroxisome proliferator-activated receptor.
